# Resilience to drought of dryland wetlands threatened by climate change

**DOI:** 10.1038/s41598-020-70087-x

**Published:** 2020-08-06

**Authors:** Steven G. Sandi, Jose F. Rodriguez, Neil Saintilan, Li Wen, George Kuczera, Gerardo Riccardi, Patricia M. Saco

**Affiliations:** 1 School of Engineering and Centre for Water Security and Environmental Sustainability, The University of Newcastle, Callaghan, Australia; 2 Department of Environmental Sciences, Macquarie University, Sydney, Australia; 3 Science Division, NSW Department of Planning, Industry and Environment, Sydney, Australia; 4 Department of Hydraulics and Research Council, National University of Rosario (CIUNR), Rosario, Argentina

**Keywords:** Hydrology, Wetlands ecology, Climate-change impacts, Projection and prediction

## Abstract

Dryland wetlands are resilient ecosystems that can adapt to extreme periodic drought–flood episodes. Climate change projections show increased drought severity in drylands that could compromise wetland resilience and reduce important habitat services. These recognized risks have been difficult to evaluate due to our limited capacity to establish comprehensive relationships between flood–drought episodes and vegetation responses at the relevant spatiotemporal scales. We address this issue by integrating detailed spatiotemporal flood–drought simulations with remotely sensed vegetation responses to water regimes in a dryland wetland known for its highly variable inundation. We show that a combination of drought tolerance and dormancy strategies allow wetland vegetation to recover after droughts and recolonize areas invaded by terrestrial species. However, climate change scenarios show widespread degradation during drought and limited recovery after floods. Importantly, the combination of degradation extent and increase in drought duration is critical for the habitat services wetland systems provide for waterbirds and fish.

## Introduction

Dryland biomes comprise almost 50% of Earth’s land surface and substantially contribute to global biodiversity and carbon sequestration^1^. In these environments, dryland wetlands are of key importance for regional biodiversity as they serve as habitat sanctuaries for aquatic and terrestrial biota in areas with very few resources^2,3^. Periodical flooding events regulate the ecological diversity of the system^4^ as flows deliver water, sediment, and associated nutrients to the floodplain^5^, allow fish and invertebrates to reach floodplain environments or distant waterholes^6^, and trigger breeding of waterbirds^3,7^ and fish^8^. During droughts, wetlands show resilience to limited water availability due to plant species either being drought-tolerant or able to re-establish when wet conditions return^9^. Future global climate change patterns and interdecadal variability projections indicate that droughts will be longer in dryland areas because of potential changes in weather patterns^10^, which could lead to global decreases of wetland extent, vegetation deterioration, and decreases in habitat services. Although these are recognized risks of pronounced interdecadal variability and climate change, estimates of vegetation deterioration in dryland wetlands are still uncertain, often due to oversimplified representation of flood–drought episodes and the vegetation response to these events.

Drylands around the world are expected to receive less rainfall over the next century, which will critically increase the pressure on dryland wetlands, as they will compete for water with irrigation and human and livestock consumption^10,11^. Climate variability will add to this pressure, with anomalies in weather patterns such as El Niño Southern Oscillation (ENSO) and the Interdecadal Pacific Oscillation (IPO) expected to become stronger in the future^12,13^, extending droughts^10^ and reducing vegetation productivity^14^. Semi-arid areas of eastern Australia, together with western North America and southern Africa are the regions of the world where the effects of ENSO and IPO are stronger^15^ and heavily influence drought periods^16,17^, so dryland wetlands at these locations will be particularly impacted in the future^8^. The extent of those impacts is difficult to predict because dryland wetlands are remarkably resilient, with many of their plant and animal species being able to cope with extreme conditions as a result of evolutionary traits. It is expected that these impacts would trigger from increased frequency, duration and intensity of extended dry periods. Because of this, the analysis of such environments requires the analysis of several decades, as these impacts would only be measurable in the long term.

Information on the inundation regime is fundamental to analyze vegetation health in dryland wetlands, and previous approaches have focused on remote sensing methods^18,19^ or spatially aggregated hydrological models^20^ to describe its characteristics. These approaches have limited resolution, either temporal (observation-based methods) or spatial (spatially aggregated hydrological methods), which is insufficient to link specific inundation episodes to vegetation physiological response and spatial organization. Another shortcoming of these approaches is that feedbacks between inundation and vegetation are not considered. While water availability is the main driver of the system dynamics by determining the physical habitat, vegetation changes can also impose feedbacks that affect the timing and extent of inundation^21^ as the vegetation provides hydrodynamic resistance that attenuates and delays the flow^22^. Numerical simulations of inundation patterns can be used to supplement observations and spatially aggregated models, allowing for the establishment of direct links with the vegetation response to inundation. Coupled flow-vegetation models have been successfully used to assess wetland vegetation health under current and future climate, and management strategies in coastal wetlands^22–25^ and some freshwater environments^26^. However, applications to dryland wetlands have remained a challenge due to our limited capacity to incorporate the vegetation response to extreme drought and flood conditions.

**Figure 1:**
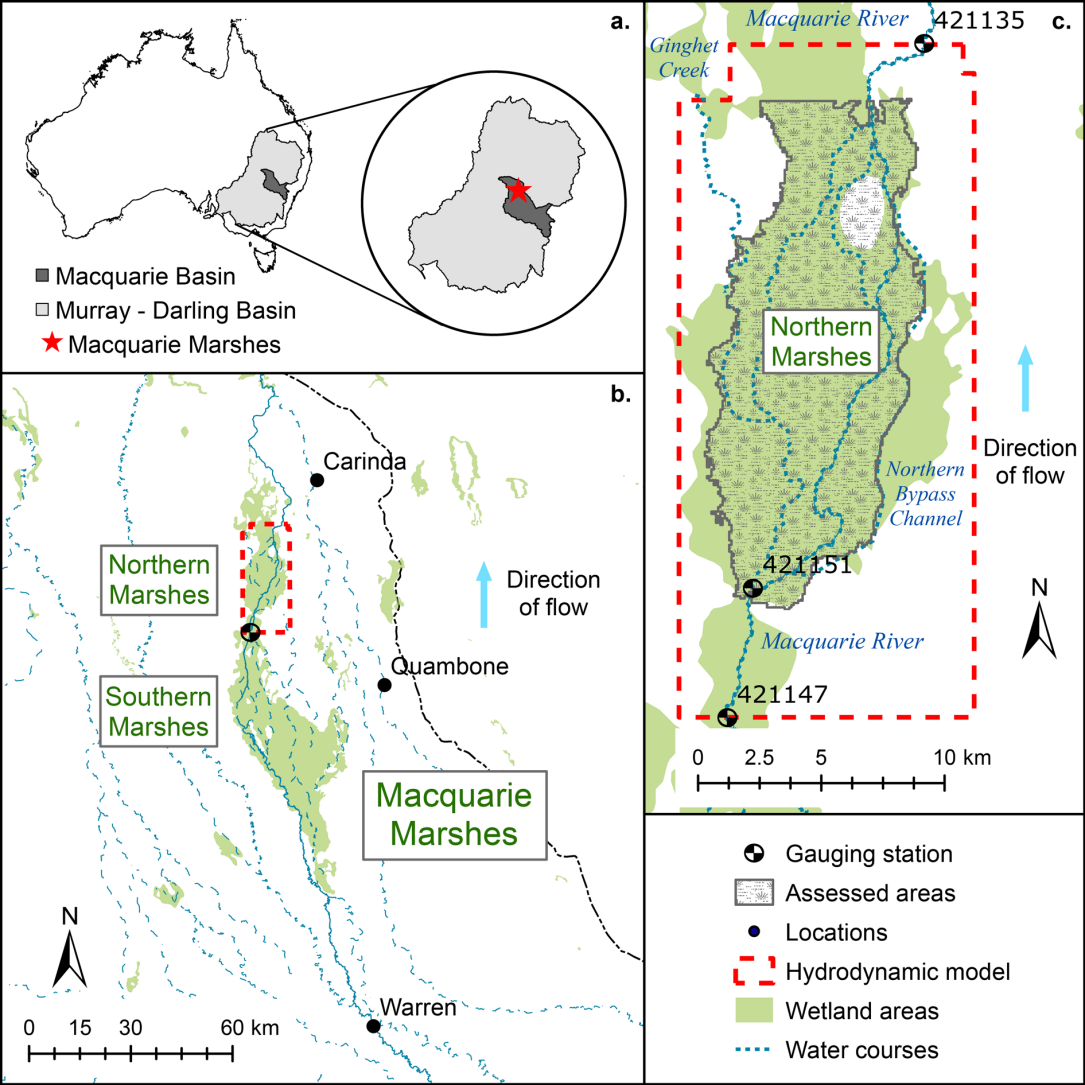
Study site. (**a**) Location of the Macquarie River Basin within the Murray–Darling Basin in Eastern Australia, (**b**) the Macquarie Marshes system within the lowland floodplain of the Macquarie River, (**c**) the Northern Marshes. Images prepared using ArcMap 10.6.1 (https://desktop.arcgis.com/en/arcmap/).

Here, we combine the use of ground vegetation surveys, remote sensing information, and continuous flood simulations to link specific inundation episodes to vegetation physiological response and spatial organization. We then use continuous simulations of flow and vegetation distribution to demonstrate that the resilience of the wetland to extreme droughts and floods can be explained by the physiological response of the wetland plants and their different strategies to cope with drought. During extended droughts grasses and reeds can die-off, but they maintain large and persistent soil seed and rhizome banks^27,28^ that allow their rapid recovery after re-wetting so they can recolonize areas invaded by terrestrial species^29^. In turn, trees resist drought by losing leaves and branches to minimize water use and rapidly recover when floods return. In the future, those traits may not be sufficient to withstand the effects of climate change if water-stressed conditions intensify and extend for longer periods. We use as a case study the Northern Marshes of the Macquarie Marshes (Fig. 1), a 225 km^2^ dryland floodplain wetland in south-east Australia with a climate heavily influenced by ENSO and IPO and expected to become dryer in the future^30^. We first investigate wetland evolution in response to climate variability, using a recent 22-year period (1991–2013) that includes one of the worst droughts on record (Millennium Drought) and some extreme flooding events. We then investigate the combined effects of climate variability and change by simulating wetland evolution for a 22-year period at the end of the century. We find that under recent climatic variability, vegetation deterioration occurred in almost half the total wetland extent during the drought, followed by a significant recovery after the drought due to floods. Projections of future climate and variability can negatively affect wetland conditions increasing the extent of deterioration by 50% during drought, with only a partial recovery after the drought due to floods. Projections also indicate that the deteriorated conditions in many wetlands areas occur over much longer periods, compromising population processes of plants, fish and birds. Our approach allows quantifying vegetation deterioration, which critically reduces the ecological services of the wetland in terms of habitat for birds, fish, invertebrates and overall wetland primary productivity.

## Results

### Wetland response to current climate variability

In order to assess the effects of successive floods and droughts typical of semi-arid Australia, we use our model to simulate the vegetation dynamics for the period 1991–2013. We selected this period because it includes a very significant series of dry and wet conditions influenced by ENSO and IPO^31^. For example, high flows observed in the years 2000 and 2010 occurred during strong positive Southern Oscillation Index (SOI—La Niña) and negative IPO periods, whereas drought conditions during 2001–2009 (Millennium Drought) were associated with a negative SOI (El Niño) (Supplementary Fig. S1). Another important reason for selecting this period was the availability of vegetation information from 1991, 2008 and 2013^32^ which we use for model setup and verification (see “Methods”). Information for these years was critical to capture the vegetation dynamics of the wetland: 1991 presented healthy vegetation conditions as a result of wet years in 1989 and 1990, 2008 evidenced considerable degradation due to the Millennium Drought, and 2013 showed vegetation recovery after another wet period.

As indicated in the “Methods” section, our vegetation model includes a woody wetland vegetation association (River Red Gum—*Eucalyptus camaldulensis*), two non-woody wetland vegetation associations (Common Reed—*Phragmites australis* and Mixed Marsh/Water Couch—*Typha* sp., sedges, *Phragmites australis*, *Paspalum distichum*) and a terrestrial vegetation association (Chenopod—*Chenopodiaceae*). In our model, non-woody wetland vegetation and terrestrial associations can transition among each other but we excluded transitions to the woody wetland association, as they did not occur in our site during the period of analysis. The woody wetland association can transition among good, intermediate and poor conditions. The definition of these conditions is linked to on-site observations of apparent mortality of trees and the composition of the understory. Healthy non-woody wetland associations occupy the understory of good and intermediate condition woody wetland patches, while the terrestrial association occupies the understory of poor condition woody patches. We use detailed information on a small number of vegetation patches to determine transition conditions^33^ and then apply those results to the rest of the wetland to simulate the extent of the vegetation over the period of analysis (see “Methods”) as a function of changing hydrological conditions.

**Figure 2:**
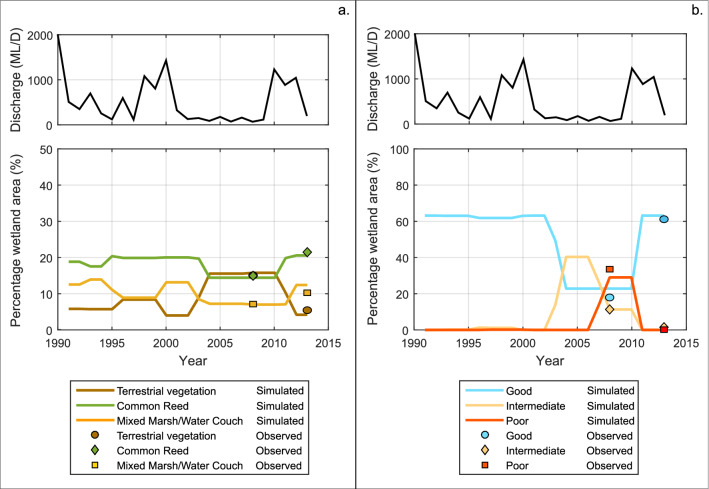
Simulated wetland vegetation extent and condition from 1991 to 2013. (**a**) Non-woody wetland and terrestrial species, (**b**) woody wetland species. Observed vegetation extent for each association in 2008 and 2013 are included in the plot to illustrate the performance of the model. Discharge series are also included for reference to dry and wet years. Plots created using MATLAB R2018b (https://au.mathworks.com/) and edited using Inkscape 0.92.4 (https://inkscape.org/).

**Figure 3:**
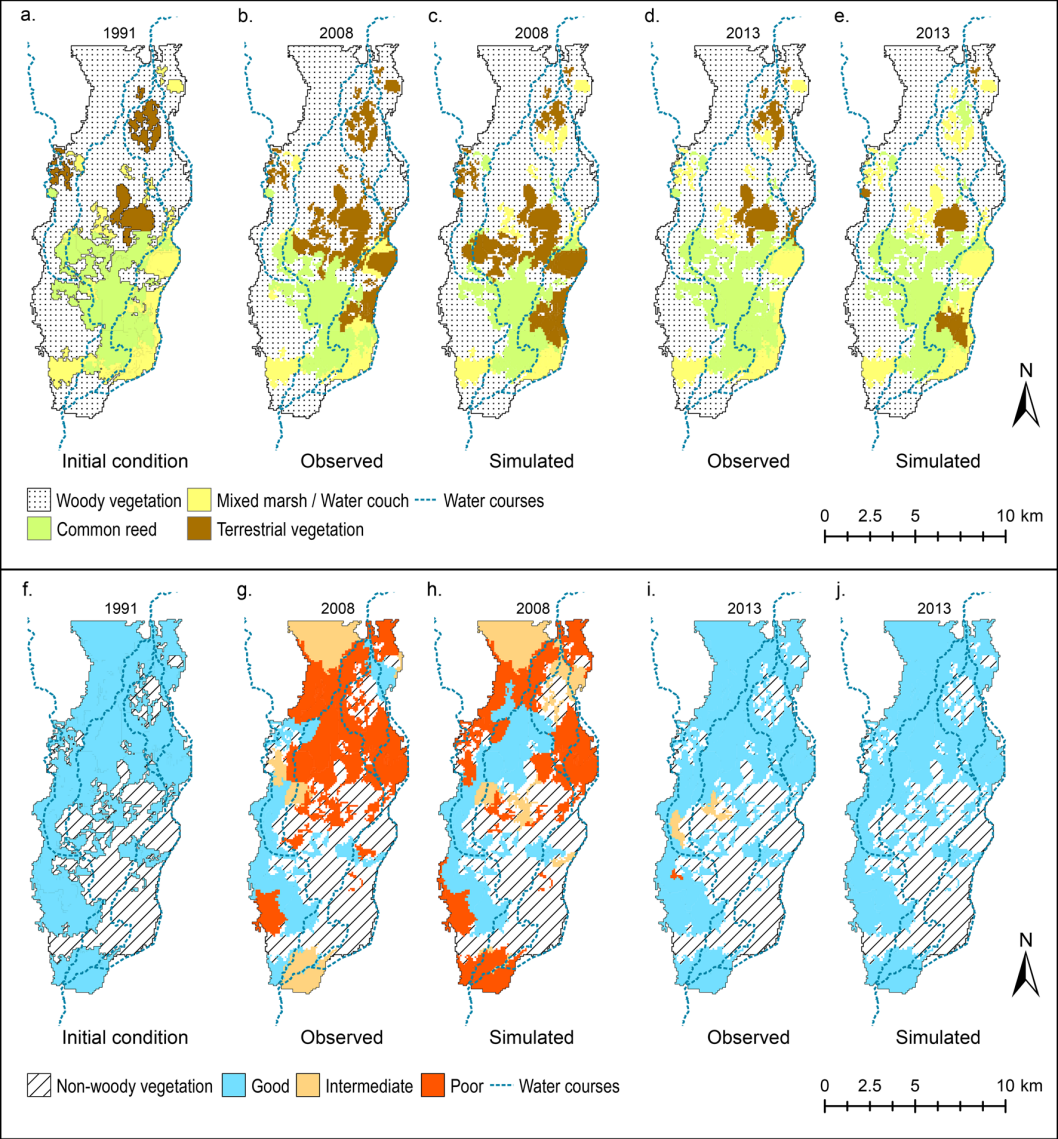
Comparison of observed and simulated vegetation extent and condition. (**a**) Initial non-woody wetland and terrestrial associations in 1991, (**b**,**c**) observed and simulated non-woody wetland and terrestrial associations in 2008, (**d**,**e**) observed and simulated non-woody wetland and terrestrial associations in 2013, (**f**) initial woody wetland association conditions in 1991*, (**g**,**h**) observed and simulated woody wetland association conditions in 2008, (**i**,**j**) observed and simulated woody wetland association conditions in 2013. Images prepared using ArcMap 10.6.1 (https://desktop.arcgis.com/en/arcmap/). Note: * 1991 woody vegetation condition is assumed to be good across the Northern Marshes after the large floods of 1989/90.

Our model is able to reproduce the observed vegetation distribution trends of wetland deterioration from 1991 to 2008 and a posterior recovery from 2008 to 2013 in terms of both total extent of each association (Fig. 2) and its spatial distribution (Fig. 3). Comparison of simulated woody and non-woody vegetation extent against observations indicates that differences between our simulation and observed extent are less than 5% of the total wetland extent (Fig. 2). The model also provides a good prediction of the general vegetation spatial patterns and their dynamics (Fig. 3). Overall spatial accuracies i.e. the ratio of correctly predicted areas and the total area of non-woody vegetation simulations are 74% and 80% for 2008 and 2013 respectively (Fig. 3c,e). The predictions of the woody association condition in 2008 are less accurate in terms of spatial distribution, with 62% overall spatial accuracy (Fig. 3h). This might be related to the assumed initial conditions of woody vegetation for 1991. In the absence of detailed information, the initial condition for woody wetland vegetation in 1991 was assumed to be good across the site (Fig. 3f) based on the fact that the previous two years (1989 and 1990) had substantial floods due to extraordinary flows. In 2013, the overall spatial accuracy of simulated non-woody vegetation was 97% as the model predicts full recovery of all woody vegetation patches (Fig. 3j) and almost all woody vegetation was reported to have recovered in the study site (Fig. 3i).

The simulations show that non-woody and woody wetland associations responded differently to climatic variability during the period of analysis. Figure 2a indicates that non-woody wetland vegetation experienced deterioration followed by a rapid recovery in response to dry and wet years in the period previous to the main drought that started in 2001. Vegetation dynamics before the drought are characterized by an initial transition from Common Reed to Mixed Marsh/Water Couch as flows receded after the big floods of 1990, followed by terrestrial invasion on Mixed Marsh/Water Couch after low flows in 1995 and a rapid recovery back to Mixed Marsh/Water Couch coinciding with high flows during 1998–2000. During the Millennium drought that started in 2001, terrestrial vegetation invaded considerable areas of both Common Reed and Mixed Marsh/Water Couch. Invasion of Mixed Marsh/Water Couch occurred in 2003 and invasion of Common Reed occurred subsequently in 2004. Both non-woody wetland vegetation associations recovered after the drought: Common Reed recovered one year after the floods of 2010 and Mixed Marsh/Water Couch recovered 2 years after the floods. However, Mixed Marsh/Water Couch recovery was not complete as some areas of Mixed Marsh/Water Couch transitioned to Common Reed.

Regarding the woody wetland vegetation dynamics, our model indicates that substantial changes only occurred during the Millennium drought (Fig. 2b). River Red Gum patches started to deteriorate in 2003, 2 years after the beginning of the drought. This deterioration resulted in more than half of the River Red Gum areas heavily impacted by the drought, with 64% of the initially good condition patches (less than 40% apparent mortality) reaching intermediate health conditions by 2004 (between 40 and 80% apparent mortality). By 2008, during the peak of the drought, 46% of the River Red Gum patches reached poor health conditions (more than 80% apparent mortality). The woody wetland vegetation rapidly recovered one year after the flood of 2010.

The spatial patterns of vegetation distribution (Fig. 3) show the typical vegetation gradient of dryland wetlands, where Common Reed is at the edge of the semi-permanent watercourses, Mixed Marsh/Water Couch is further away and River Red Gum occupies areas with less frequent inundation^9^. The Northern Marshes present a large central Common Reed concentration clustered around a flat area where the main watercourses are shallow and frequently inundate the floodplain (1991 distribution). This central area is generally surrounded by Mixed Marsh/Water Couch, which is in turn surrounded by River Red Gum. During the drought (2008 distribution), flows tended to concentrate on the western side of the Marshes, resulting in substantial wetland deterioration on the eastern side with extensive dryland vegetation invasion and River Red Gum defoliation (Fig. 3b,g). After the drought (2013 distribution), almost all the wetland vegetation regained its pre-drought extent, except for a few patches where Mixed Marsh/Water Couch was replaced by Common Reed.

### Wetland response to climate change

Climate-driven changes in streamflow over the next century are predicted to be considerable in semi-arid ecosystems of the Australian Central Slopes, where the Macquarie Marshes are located. The combination of decreases in annual rainfall and increases in evapotranspiration will result in substantial streamflow reductions^30,34^. Regarding climate variability, some evidence suggests the strengthening of ENSO weather patterns with potential for an intensification of El Niño driven droughts^12^.

In order to assess the potential combined effects of climate variability and change, we incorporate information on projected climate change in the Macquarie Marshes into our simulations. We use runoff projections for the end of the century considering the Representative Concentration Pathway scenario 8.5 (RCP8.5) from the Intergovernmental Panel on Climate Change (IPCC), Fifth Assessment Report (AR5), which result in a runoff change of − 20% (± 40%)^30,34^ (see “Methods”). We run three scenarios with our model using synthetic series of discharges corresponding to the median value and the upper and lower limits of the projections. Accordingly, we adjust our original discharge series (current scenario) using proportionality factors of 0.8, 1.2 and 0.4, representing the median, upper and lower limits of the projections, respectively. These factors affect the magnitude of discharges during flood and drought periods so our results for the median and lower limit projections can account for an intensification of drought conditions including duration^12^. The previous projections do not consider changes in the frequency of drought events as projections of rainfall intensity and seasonality were excluded from the analysis^30^.

**Figure 4:**
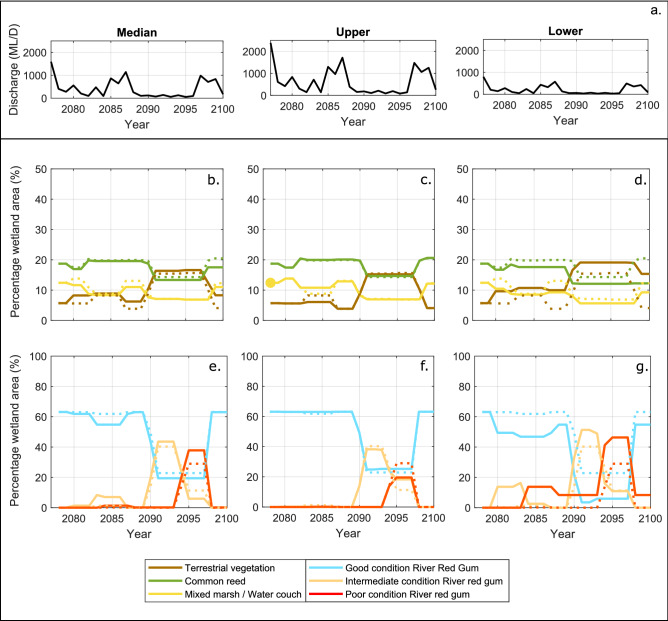
Simulated vegetation extent and condition under different climate change scenarios. (**a**) Discharge series for the Median, Upper and Lower limit scenarios; (**b**–**d**) simulated non-woody wetland and terrestrial vegetation extent under Median, Upper and Lower limit scenarios; and (**e**–**g**) simulated woody wetland association condition under Median, Upper and Lower limit scenarios. Dotted lines represent simulated results for the Current scenario. Plots created using MATLAB R2018b (https://au.mathworks.com/) and edited using Inkscape 0.92.4 (https://inkscape.org/).

Our projections indicate that wetland vegetation will not be drastically affected by moderate changes in the inundation regime corresponding to the Median scenario (− 20% runoff) and Upper limit scenario (+ 20% runoff); however, strong wetland deterioration will occur for the considerably dryer conditions corresponding to the Lower limit scenario (− 60% runoff) (Fig. 4). Compared to the Current scenario, the Median scenario will result in more areas of Mixed Marsh/Water Couch being encroached by terrestrial vegetation (Fig. 4b) and a moderate degradation in River Red Gum health condition (Fig. 4e) during the first half of the simulation period. In the second half of the simulation period, during the simulated extended drought, wetland conditions are projected to be similar to those of the Current scenario, but with more River Red Gum patches degrading to poor conditions. Recovery of the vegetation after the drought is projected to be strong as in the Current scenario, but there will be areas of terrestrial vegetation that will not transition back to Common Reed and Mixed Marsh/Water Couch. For the Upper limit scenario, even though the projections correspond to a 20% increase in discharges, they do not show a significant improvement of wetland health and extent compared to the Current scenario (Fig. 4c,f).

**Figure 5:**
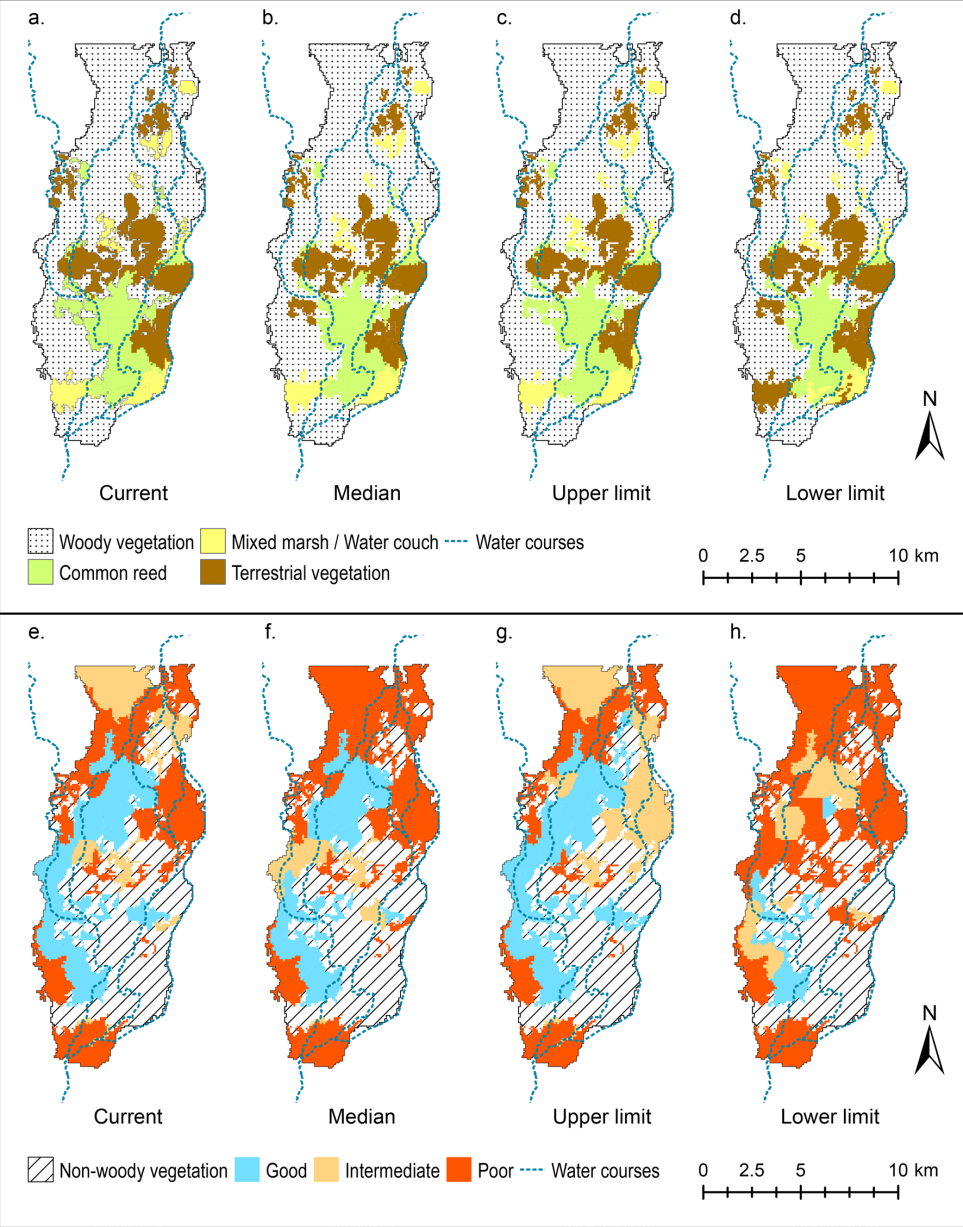
Vegetation distribution and condition at the peak of the drought. (**a**–**d**) Non-woody wetland and terrestrial associations at the peak of the drought under Current, Median, Upper and Lower limit scenarios, (**e**–**h**) woody wetland associations condition at the peak of the drought under Current, Median, Upper and Lower limit scenarios. Images prepared using ArcMap 10.6.1 (https://desktop.arcgis.com/en/arcmap/).

Projections of the spatial distribution of the vegetation for the Median scenario indicate that areas to the east of the core wetland that were not affected in the Current scenario will start to deteriorate, with more Common Reed patches (29%) transitioned to terrestrial vegetation and more apparent tree mortality in River Red Gum patches (60%) projected at the peak of the drought (Fig. 5b,f). This trend intensifies for the Lower limit scenario, which shows dramatic deterioration in areas south of the core wetland due to further losses of Mixed Marsh/Water Couch and in the north more severely stressed River Red Gum trees (Fig. 5d,h). The projected advance of terrestrial vegetation on non-woody wetland vegetation and deterioration in River Red Gum areas in the Median and Lower scenarios will effectively result in a contraction of the core wetland extent. The Lower limit projections indicate potentially catastrophic changes for the wetland (Fig. 4d,g). The 60% reductions in discharge will trigger a widespread replacement of non-woody wetland vegetation by terrestrial vegetation which will occur over the entire period of the projections. During the drought, 43% of the original non-woody wetland vegetation will be replaced by terrestrial vegetation. Terrestrial vegetation invasion will be accompanied by a continuous deterioration of the River Red Gum areas, with 73% of River Red Gum reaching poor conditions at the peak of the drought. In the Lower limit scenario, the wetland will partially recover after the drought, but some of the vegetation losses will be irreversible (31% deteriorated non-woody vegetation and 13% deteriorated River Red Gum remaining at the end of the simulation). As already mentioned, increased inundation corresponding to the Upper limit scenario will not represent substantial changes on wetland extent and condition during the peak of the drought. Compared to the Current scenario, the Upper limit scenario will result in a slight improvement of River Red Gum health on patches to the north of the core wetland (Fig. 5c,g).

## Discussion

Our results for the Current scenario show that dryland wetland vegetation has a remarkable capacity to adapt to climate variability. River Red Gum trees can tolerate many years without water and can rapidly regenerate after floods, while non-woody wetland vegetation reduces its extent during droughts but immediately respond to re-wetting by seed or rhizome regeneration^29^. This resilience depends on periodic floods^4^, as riverine and floodplain species rely on longitudinal and lateral connectivity for maintenance^35,36^. Modelling indicates that the large floods during the 1990s produced considerable inundation of the floodplain and consequently healthy conditions across the marshes, which were maintained through a series of years with variable discharges until the Millennium drought in the 2000s (Figs. 2, 3) in agreement with observations^32^. At the peak of the drought (2008), the extent of healthy wetland vegetation (Common Reed, Mixed Marsh/Water Couch and River Red Gum in good and intermediate conditions) reduced to almost half of the extent in 1991. Those extreme conditions prevailed for a period of 3 years (2008–2010). A period of floods after the drought generated almost complete wetland recovery by 2013.

**Figure 6:**
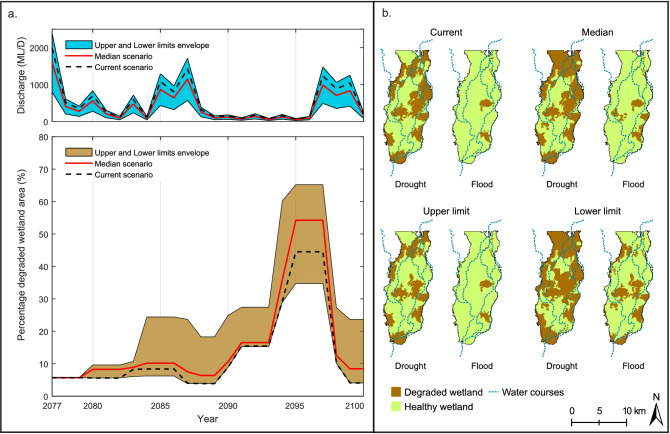
Wetland vegetation response to drought-flood pulses under current and projected conditions. (**a**) Evolution of degraded wetland areas showing increases in extent and duration of degradation for the Median and Lower limit scenarios during drought and less recovery during flood. (**b**) Spatial distribution of degraded and healthy wetland areas showing increases in wetland contraction and loss of connectivity during drought for the Median and Lower limit scenarios. Maps prepared using ArcMap 10.6.1 (https://desktop.arcgis.com/en/arcmap/), plots created using MATLAB R2018b (https://au.mathworks.com/). Image edited using Inkscape 0.92.4 (https://inkscape.org/).

The long term changing climate, in combination with natural variations of drought and flood phases could amplify the effects on wetland vegetation. The degraded wetland areas, quantified as the percentage area of terrestrially invaded non-woody vegetation and woody wetland vegetation in poor conditions, are expected to increase under the Median and Lower limit scenarios (Fig. 6a). These two projections of reduced flow discharges indicate increases of 20% and 50% of degraded wetland at the peak of the drought, respectively when compared to the degradation for the Current scenario (Fig. 6a). The reduced discharge scenarios also show that the wetland will be less able to recover after the floods, as less patches will receive sufficient inundation for recovery after the drought period. The remaining degraded wetland area values are 10% and 25% for the Median and Lower limit scenario compared to almost no remaining degraded wetland in the Current and Upper limit scenarios (Fig. 6a). In addition, the drought-affected phase will be longer, particularly for the Lower limit scenario: 25% of the wetland will be degraded for a continuous period of 10 years, and more than half of the wetland will be degraded for five consecutive years during extreme drought conditions.

These changes in vegetation will have an impact on the habitat services, multifunctionality and biodiversity that depend on the plant species richness^37^, as loss and fragmentation of preferred habitats will happen due to reduced connectivity during drought (Fig. 6b). Some animal species will see important reductions of their preferred habitat over considerable periods of time that may affect their population. A number of native fish species (e.g. Fly-Specked Hardyhead, Bony Herring, Carp Gudgeon, Golden Perch, Freshwater Catfish, Australian Smelt, Silver Perch and Spangled Perch) use the floodplain wetlands rather than the main channel as preferred habitat^8,38^, so they would have to adapt to the use of a reduced wetland area for refugia^38^ and their populations will decrease. Comparing our projections for the Lower limit scenario with the Current scenario, we can expect that the extent of wetland area usable by fish (Common Reed and Mixed Marsh/Water Couch) will reduce by about 25% both during drought and flood periods (Fig. 4d) due to terrestrial encroachment. We can also expect that drought conditions in Common Reed and Mixed Marsh/Water Couch areas will last longer. Even the native fish that prefer the main channel (e.g. Murray Cod) rely on floodplain inundation for suitable spawning habitat, and long periods without flooding will compromise their reproductive cycle^8^. In particular, fish species with short life-spans (i.e., Bony Herring and Spangled Perch) can be locally extinct in wetland areas with no flooding for more than 3 years. In contrast, introduced fish species that mainly spawn in the main channel (i.e., Carp, Gambusia, and Goldfish)^8^, but use the floodplain opportunistically, will not be substantially affected by the reduction of wetland areas and will potentially out-compete the native fish population.

The preferred habitat of waterbirds will also see important changes, particularly regarding breeding and refugia. Colonial-nesting waterbirds and ducks require well-flooded areas near their nests to commence breeding and they have very specific requirements in terms of vegetation types^39^. A large number of waterbirds (e.g. Little Pied Cormorant, Little Black Cormorant, Cattle Egret, Little Egret, Great Egret, White-Necked Heron, Rufous Night Heron, Yellow-Billed Spoonbill, Royal Spoonbill, Australian White Ibis) prefer to nest and breed in healthy River Red Gums^8^ with sustained inundation under trees^39^, so the considerable reduction woody vegetation with good and intermediate health will directly impact their population. River Red Gum breeding habitat during drought will decrease by 30% and 90% of the current extent for the Median and Lower limit scenarios, respectively. Even though the maximum extent of breeding habitat during flood periods will not change for the Median scenario and will only decrease by 15% for the Lower limit scenario, the longer duration of degraded conditions in large parts of the wetlands will result in less frequent breeding events.

Other effects of vegetation deterioration are less obvious, for example, disruptions to primary sources of the food chain due to losses of woody wetland vegetation. Fresh leaf shedding and litter-fall from River Red Gum trees occur as a response to flooding events, providing the floodplain with nutrients^40^ and dissolved organic carbon^41^. More areas of River Red Gum with poor conditions projected for the Median and Lower limit scenario will result in less fresh organic matter to serve as food sources for aquatic organisms or to help support the production of the understory wetland vegetation. In fact, the non-woody understory wetland vegetation relies on overstory woody wetland species like River Red Gum for coverage^29^. Vegetation deterioration can also lead to a decrease in soil carbon sequestration. In Common Reed patches, soil carbon sequestration is linked to the frequency of inundation^42^. Shifts towards drier conditions will result in less inundation of the non-woody wetland vegetation, which will not be able to fully recover resulting in more carbon loss to the atmosphere.

Other mechanisms, which are out of the scope of this contribution, can also negatively affect the wetland vegetation and could potentially lead to more pronounced deterioration in the future. For example, invasive species have been identified in the study site and other dryland wetlands of Australia^43,44^. Similarly to native species, invasive species are flood dependent and sensitive to altered water regimes. Under dryer conditions, native vegetation can potentially be displaced or dominated by invasive species^45^ and there are limits to the capacity of seedbanks and rhizomes to survive extended dry periods^46^. The climate change scenarios explored in this research show two cases (Median and Lower limit scenarios) where invasive species could have potential impacts on the native vegetation or where the vegetation could not recover from seedbanks or rhizomes. In addition, water management upstream of the site has not been incorporated directly into our analysis. Climate change could trigger the increase of upstream water abstractions, which would exacerbate the impacts of climate change on the wetland vegetation that we have presented here. One final limitation of our results is that we have not included specific functions representing local facilitation mechanisms such as seed or rhizome banks, soil moisture, and resource re-distribution. The explicit inclusion of such functions would require significantly more spatial information and data. Our simulations,however, are carried over the patch scale where the water regime is considered as the main global driver of vegetation and can indirectly integrate local drivers that occur at a scale smaller than the patch or the cell^47^.

Results presented here provide quantifiable projections of wetland deterioration that allow for an analysis of the wetland resilience under different climate change scenarios. These results are generated for an iconic wetland in semiarid Australia, and the framework integrates the inundation regime with available information of vegetation response to flood and drought. Including the inundation regime in the analysis of different ecosystems is of major importance for the management of environmental water and restoration^36^. This framework can be extrapolated to similar environments, which will require the study of the response of the targeted vegetation under different inundation regimes using a similar methodology. The development of models that can realistically predict the extent of wetland vegetation presents a stepping-stone for analyzing habitat services for fish, migratory birds, and also to analyze carbon sequestration.

## Materials and methods

### Modelling approach

Spatially distributed hydrodynamic information on water levels is used to compute characteristics of the inundation regime (water depth, duration, and frequency) associated with vegetation establishment/survival/deterioration. Our main assumption is that local surface water dynamics and floodplain hydrological connectivity^48–51^, imposes a gradient of conditions for vegetation establishment across the floodplain^9^, which gives rise to a patch-like distribution of different plant species^9,50,52^. This distribution of vegetation is dynamic, as wetlands expand as a response to flood pulses^53^ and contract during droughts. Our vegetation response relations are based on ground and remote sensing observations of vegetation health conditions and the corresponding inundation regime associated with healthy and deteriorated patches of different vegetation associations^33^. These relations allow us to predict vegetation distribution on an annual basis using plant-specific information on critical inundation conditions for vegetation establishment and survival.

### Calculation of the inundation regime

We quantify the inundation regime over time with the use of a quasi-two-dimensional hydrodynamic model. This model is built on a spatially distributed cell-scheme that solves hydraulic and hydrological relations between cells using a finite differences scheme. For each time step, mass conservation between cells is solved with a fully two-dimensional scheme and momentum conservation between cells is solved using simplified one-dimensional formulations. During each iteration, the model solves mass conservation as a function the variation in water elevation over time:

1 $$A_{Si} \frac{{dz_{i} }}{dt} = \mathop \sum \limits_{k = 1}^{j} Q_{k,i} ,$$

where *A*_*Si*_ is the surface wetted area and *z*_*i*_ is the surface elevation in the cell *i*, and *Q*_*k,i*_ are discharges between cell *i* and the *j* surrounding cells. Momentum conservation is used to solve for *Q*_*k,i*_. The simplification of the momentum conservation relationship depends on the type of cell because the cells can represent the river network or the floodplain. For example, for a link between two cells (*i* and *k*) located in the floodplain, the discharge between the cells can be estimated with the use of Manning’s formula:

2 $$Q_{k,i} = \frac{{A_{k,i} R_{k,i}^{2/3} }}{{n_{k,i} }}\left( {\frac{{z_{k} - z_{i} }}{{x_{k} - x_{i} }}} \right)^{1/2} ,$$

where *A*_*k,i*_ , *R*_*k,i*_ , and *n*_*k,i*_ are the averaged cross-sectional area, hydraulic radius and Manning roughness coefficient between the two cells. The term *x*_*k*_*–x*_*i*_ estimates the distance between the centroids of the cells. The resulting differential equation system is solved with the Gauss–Seidel iterative method and model stability is assured with the Courant–Friedrichs–Lewy condition. Stability is achieved by using numerical iteration time-steps that varied from 1 to 5 s.

This spatially distributed hydrodynamic model has been previously used in studies of riverine floodplains^54,55^ and coastal wetlands^22,25^. Initial assessment of model implementation for our study site showed that the performance of the model is similar to a fully integrated two-dimensional model^56^. Calibration of the model^33^ consisted on obtaining the combination of Manning roughness (Supplementary Table S1) for vegetated substrates and watercourses that best predicted water depth and inundation extent when compared against water depth time-series recorded at gauging station No.421151 (Fig. 1c), obtained from WaterNSW (https://realtimedata.waternsw.com.au/), and inundation maps obtained from satellite imagery^57^. Model performance was assessed using three indices for water depth predictions (percent bias, Nash–Sutcliffe coefficient and ratio of the root mean square error to the standard deviation of measured data) and two indices for inundation extent (Overall Accuracy and Cohen’s κ). Results of the calibration and subsequent blind model testing using calibrated roughness values showed excellent model performance, and have been included in the Supplementary Material (Supplementary Figs. S2 and S3, Supplementary Table S2).

The model domain consists of a rectangular grid cell with a resolution of 90 × 90 m, with a total of 40,096 cells. As often occurs when modelling large domains for long periods of time, the resolution was a compromise between the level of detail and the computational cost^58^. Each cell of the model contains topographic elevation [obtained from resampling a 1 m resolution LiDAR Digital Elevation Model (DEM) provided by NSW Office of Environment and Heritage], characteristics of the floodplain (vegetation associations, Manning roughness), and characteristics of channels and streams (cross-sections, stream channel roughness). Model upstream inputs consist of water levels in the Macquarie river recorded at gauging station No. 421147 (Fig. 1c), and the downstream boundary condition is set using water levels recorded at station No. 421135 (Fig. 1c), extended over the whole downstream boundary in order to represent the diffused floodplain flow observed. All other boundary conditions are set as no-flow. The input and output data have a daily time-step.

### Definition of vegetation patches

The analysis and simulation of vegetation response to the inundation regime is carried out at the patch scale. Vegetation in dryland wetlands tends to group in stands of dominant vegetation associations forming a mosaic-like distribution that responds primarily to the inundation regime but also to local facilitation mechanisms^47^ as also observed in other wetland systems^27,29^. In our study, we defined 110 patches based on the observed vegetation dynamics and considering spatial features that could potentially affect vegetation response like distance to streams. We used surveyed vegetation distributions for 1991 (wet conditions) and 2008 (dry conditions)^32^ to define patches that could capture the observed flood-drought vegetation dynamics but we also further subdivided large patches into riparian and floodplain patches as they may behave differently under future conditions. We also merged small patches with observed similar dynamics in order to reduce bias in the estimation of patch integrated inundation values from the hydrodynamic model. Our final patch distribution included patches ranging in size from 0.16 km^2^ (20 cells of the hydrodynamic model) to 6.8 km^2^ (840 cells of the hydrodynamic model). This patch distribution of the vegetation is consistent with other studies of wetland systems in Australia^59–62^ and the US^26^.

### Minimum Inundation Index (MII)

We use the Minimum Inundation Index (MII)^33^ as a descriptor of the inundation experienced by a vegetation patch in a year. The MII is defined as the percentage area of the patch with annual inundation conditions that meet minimum water requirements of the specific vegetation in terms of water depth and flood duration. Inundation conditions are obtained from the hydrodynamic simulation results, while minimum water requirements are defined based on the literature for each vegetation association (Supplementary Table S3). For example, in our final simulations, we consider Common Reed requires about four months (33% of the year) of inundation above 0.02 m, while Mixed Marsh/Water Couch and River Red Gum can cope with 3 months (25% of the year) of inundation above 0.02 m. For every cell in a patch, a water depth versus flood duration curve is calculated and compared to the minimum water requirements. The number of cells with inundation conditions at or above the minimum water requirements is converted to a percentage area of the patch to determine the value of MII. For example, in a hypothetical five-cell patch where three of the five cells comply with the minimum water requirements, the resulting MII value is 60% (Supplementary Fig. S4).

### Threshold selection for wetland vegetation response

To obtain vegetation transition thresholds based on the MII values, we selected a number of vegetation patches that remained healthy during the Millennium Drought, as well as patches that either transition to terrestrial vegetation (in the case of non-woody wetland associations) or severely deteriorated (in the case of the woody wetland association) and we followed the conceptual model presented in Supplementary Fig. S5. Time-series of MII describe the inundation regime of the selected patches, which can be used to identify the MII thresholds associated with transition to terrestrial vegetation or severe deterioration. The observed vegetation dynamics in our study site indicated that non-woody wetland vegetation could transition to other non-woody wetland vegetation or terrestrial vegetation, while woody vegetation mainly suffered deterioration instead of transition to other associations^32^. The frequency of inundation is another inundation characteristic required for vegetation survival (Supplementary Tables S3 and S4) which defines transitions and helps identify MII thresholds. For instance, for non-woody wetland associations (Common Reed and Mixed Marsh/Water Couch), a series of three consecutive years below the MII threshold (frequency of 0.33) leads to a transition to terrestrial association^63^ (Supplementary Table S3). For the woody vegetation patches, the health condition can be related to values of inundation frequency above the minimum requirements, as reported in the literature^63,64^ (Supplementary Table S4). River Red Gum can survive anywhere from one to ten consecutive years without minimum inundation^65^, however, health conditions will gradually deteriorate. Based on the literature^33^ we assumed that more than two years below the MII threshold will result in transition, from good to intermediate conditions and more than 6 years below the threshold will trigger transition to poor conditions (Supplementary Table S1).

The surveyed vegetation distributions^32^ can be used to define patches of each vegetation, but cannot be used to detect transitions, due to their low temporal resolution. In order to have a continuous indication of vegetation health and detect transitions that can be used to define the MII thresholds, we used green Seasonal Fractional Cover (SFC)^66^, which is a remote sensing estimation of ground cover developed using a combination of Landsat imagery and field measurements by the Joint Remote Sensing Research Program. This database provides seasonal averaged fractions of green, non-green and bare soil cover with a resolution of 30 × 30 m, from which we select autumn green SFC values as representative of vegetation health at the end of the growing season. The selection of the green SFC allows for an assessment of the decay of the vegetation due to consecutive years with inundation values below the MII thresholds^33^. By analyzing patches that remained healthy during the drought and patches that transitioned according to the 1991 and 2008 surveys, SFC analysis allowed to pinpoint the transitions within that period, which included transitions of non-woody wetland vegetation to terrestrial vegetation and different levels of deterioration of woody vegetation^32^. The transitions occurred between 2003 and 2006 as a result of severe dry conditions during the Millennium Drought (Supplementary Fig. S6). The same pattern was observed in many other sites across the region^29^. For the non-woody vegetation, transitions to poor conditions were associated with terrestrial invasion of the understory, which was captured by the SFC (Supplementary Fig. S6).

With the values of MII for each patch obtained following the procedure described above and the SFC analysis of transitions, MII thresholds were obtained by trial and error in order to comply with the frequency requirements to trigger transitions (Supplementary Tables S3 and S4).

### Vegetation modelling

Using the MII thresholds and frequencies, we set up and calibrated a vegetation model that predicts changes on 110 vegetation patches of the wetland, each one containing one of six possible vegetation classes (i.e. Common Reed, Mixed Marsh/Water Couch, Terrestrial, good condition River Red Gum, intermediate condition River Red Gum and poor condition River Red Gum). We set the observed vegetation distribution in 1991 as the initial conditions and we carry out simulations over a 22-year period (1991–2013).

Using the results of the spatially distributed hydrodynamic model, the vegetation model checks if inundation transition thresholds and inter-annual inundation frequency are met within patches for every water year (June–May), using MII as a descriptor of the inundation regime. If the inundation conditions indicate that a patch undergoes transition, the Manning’s roughness (Supplementary Table S1) values are updated to reflect this change before the next water year, which provides a feedback mechanism between the flow and the vegetation. The selection of the transition thresholds was performed by selecting the value of MII from the range of possible values (Supplementary Table S5) that yields the best prediction of the vegetation extent in 2008 and 2013 when compared to the observed vegetation (Fig. 2). Selected MII thresholds values for Common Reed and Mixed Marsh/Water Couch transitions were 55% and 20% respectively, whereas a value of 55% gave the best predictions for River Red Gum reaching poor conditions. We evaluated the performance of the model by comparing the simulated extent of the non-woody wetland associations and terrestrial association against the observed distribution in 2008 and 2013^32^, and by comparing the simulated canopy condition of the woody wetland vegetation against the reported canopy conditions for the same periods.

### Climate change scenarios

For our climate change projections, we use results from an assessment of climate change in different regions of Australia carried out by CSIRO and the Bureau of Meteorology^34^. This assessment uses an ensemble of global climate models (GCMs) to predict changes in annual runoff at the end of the century from downscaled precipitation and potential evapotranspiration in the region where the Macquarie River catchment is located. The GCMs used are part of the Coupled Model Intercomparison Project Phase 5 (CMIP5), used in the IPCC AR5. Two different methods were used to downscale precipitation and potential evapotranspiration,specifically, the Bureau of Meteorology Analogue-based SDM statistical downscaling model^67^ with 22 CMIP5 GCMs used as input, and the CCAM dynamic downscaling model^68^ with six CMIP5 GCMs used as input (Table S6). Regional runoff series are then estimated by incorporating precipitation and potential evapotranspiration into a hydrologic framework^69^ and changes in annual runoff are given as percentage change (ΔR) with respect to the a 20-year average. We consider projections of annual runoff change corresponding to the greenhouse emission scenario RCP8.5. The results of the ensemble indicate a median ΔR of − 20% (20% runoff reduction) with upper limit of 20% (10th percentile) and lower limit of − 60% (90th percentile)^30^. We define three scenarios for our hydrodynamic simulations by adjusting the recent records of discharge at gaging station No. 421147 (Current scenario) using proportionality factors of 0.8 (Median scenario), 1.2 (Upper limit scenario) and 0.4 (Lower limit scenario). A similar methodology has been implemented for assessment of climate change impacts in dryland areas of Australia^20^ and the US^70,71^.

## Supplementary information

Supplementary information.

## Data Availability

Daily river water depths and discharges available from WaterNSW at https://realtimedata.waternsw.com.au/. We also acknowledge the work of the Joint Remote Sensing Research Project (JRSRP) for developing the algorithms of Seasonal Fractional Cover (SFC). SFC data available at https://data.auscover.org.au/. We acknowledge the work of CSIRO and the Bureau of Meteorology (BoM) of Australia for their work in developing climate change projections and regional cluster reports available at https://www.climatechangeinaustralia.gov.au/. Other data available at request. Special thanks: The authors would like to thank Professor Andrea Rinaldo from EPFL (CH) and Università di Padova (IT) for his feedback during the preparation of the manuscript.
