# Supplementary material for: Resilience to drought of dryland wetlands threatened by climate change
Source: Sci Rep. 2020 Aug 6;10:13232. doi: 10.1038/s41598-020-70087-x (PMC7414121; doi:10.1038/s41598-020-70087-x)
Supplement: Supplementary file 1 — Supplementary information. [file 41598_2020_70087_MOESM1_ESM.pdf]

SUPPLEMENTARY MATERIAL

Resilience to drought of dryland wetlands threatened by climate change

Authors

S. G. Sandi[1*], J. F. Rodriguez[1], N. Saintilan[2], L.Wen[3], G. Kuczera[1], G. Riccardi[4], P.M. Saco[1*]

Affiliations

[1] School of Engineering and Centre for Water Security and Environmental Sustainability, The University of Newcastle, Australia

[2] Department of Environmental Sciences, Macquarie University, Australia

[3] Science Division, NSW Department of Planning, Industry and Environment, Australia

[4] Department of Hydraulics and Research Council of National University of Rosario (CIUNR), Argentina

* Corresponding authors: Patricia M. Saco patricia.saco@newcastle.edu.au

Steven G. Sandi steven.sandirojas@newcastle.edu.au

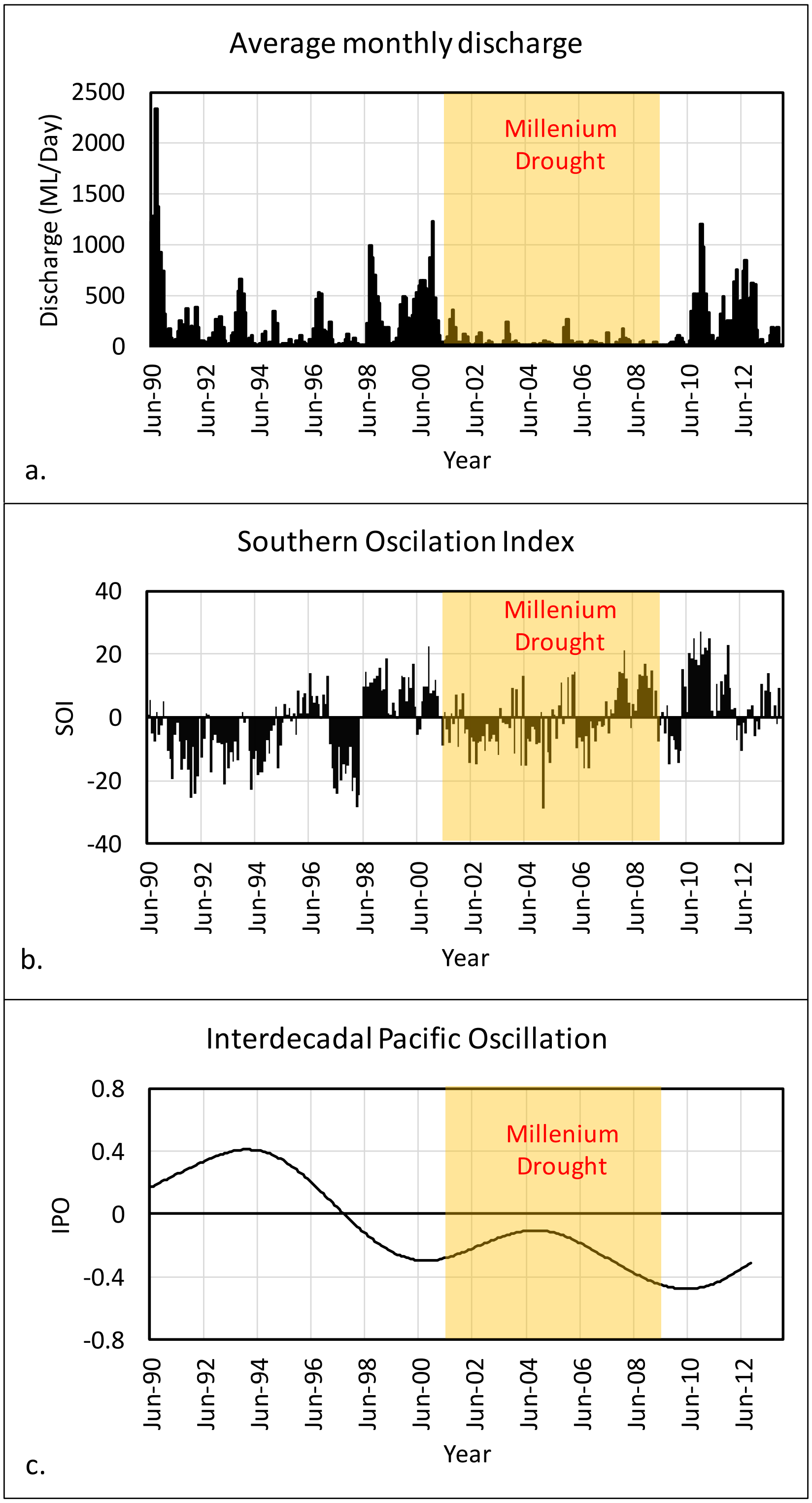

Fig. S1. Discharge and climate variables timeseries. (**a**) Average monthly discharge timeseries recorded at gaging station No.421147, (**b**) Southern Oscillation Index (SOI), and (**c**) Interdecadal Pacific Oscillation (IPO).

Note: Discharge data was adapted from WaterNSW (https://www.waternsw.com.au/). SOI data was downloaded from the Australian Bureau of Meteorology (BOM) (http://www.bom.gov.au), IPO data presented here corresponds to the Tripole Index (TPI) NOAA ERSST V5 filtered version (https://www.esrl.noaa.gov).

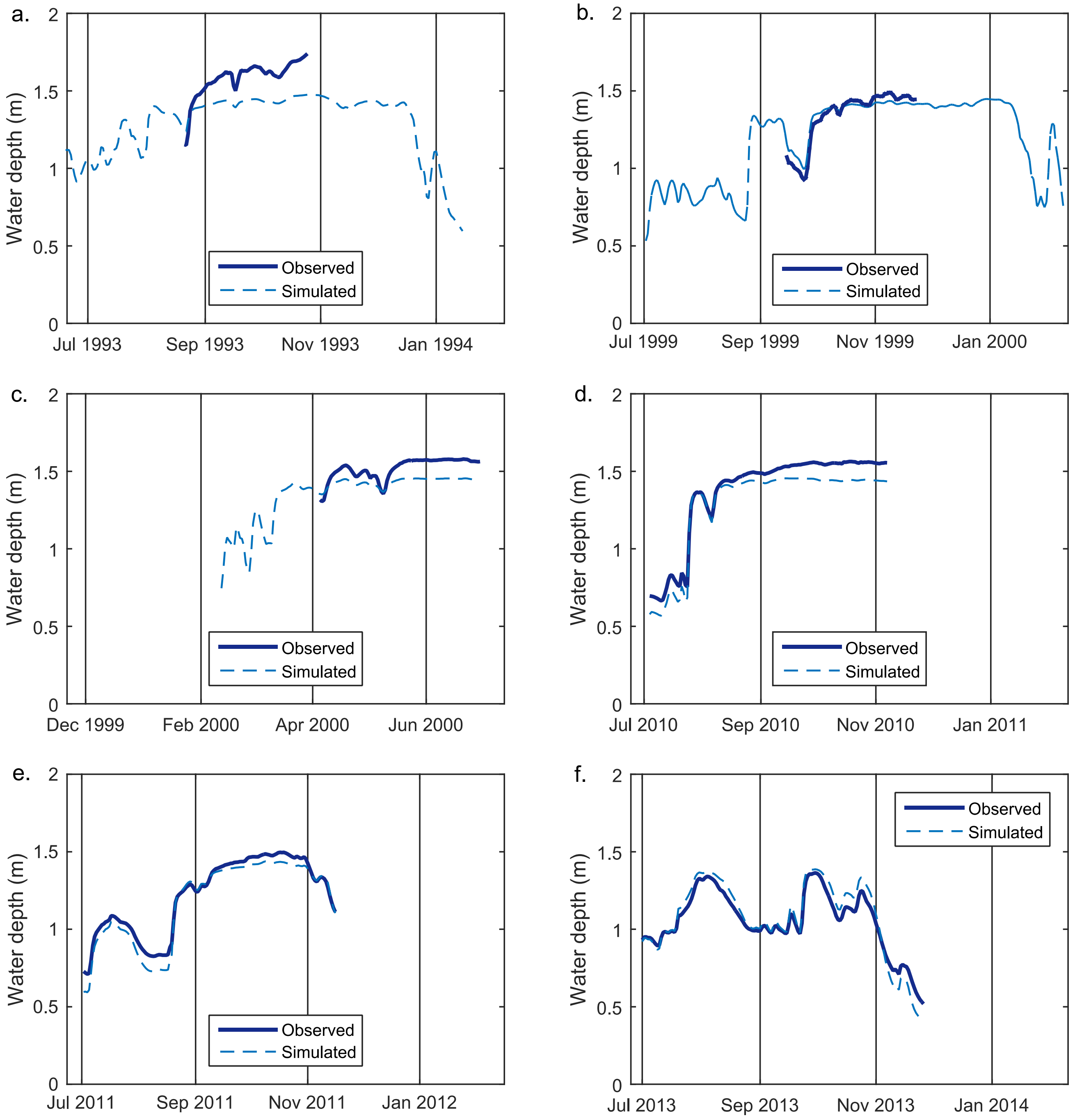

Fig. S2. Hydrodynamic model results - water depth comparison. Comparison of model results against water levels recorded at station No.421151 for six different events used for calibration and model testing. Performance indices presented in Table. S1.

Note: Adapted from Sandi et al. (2019).

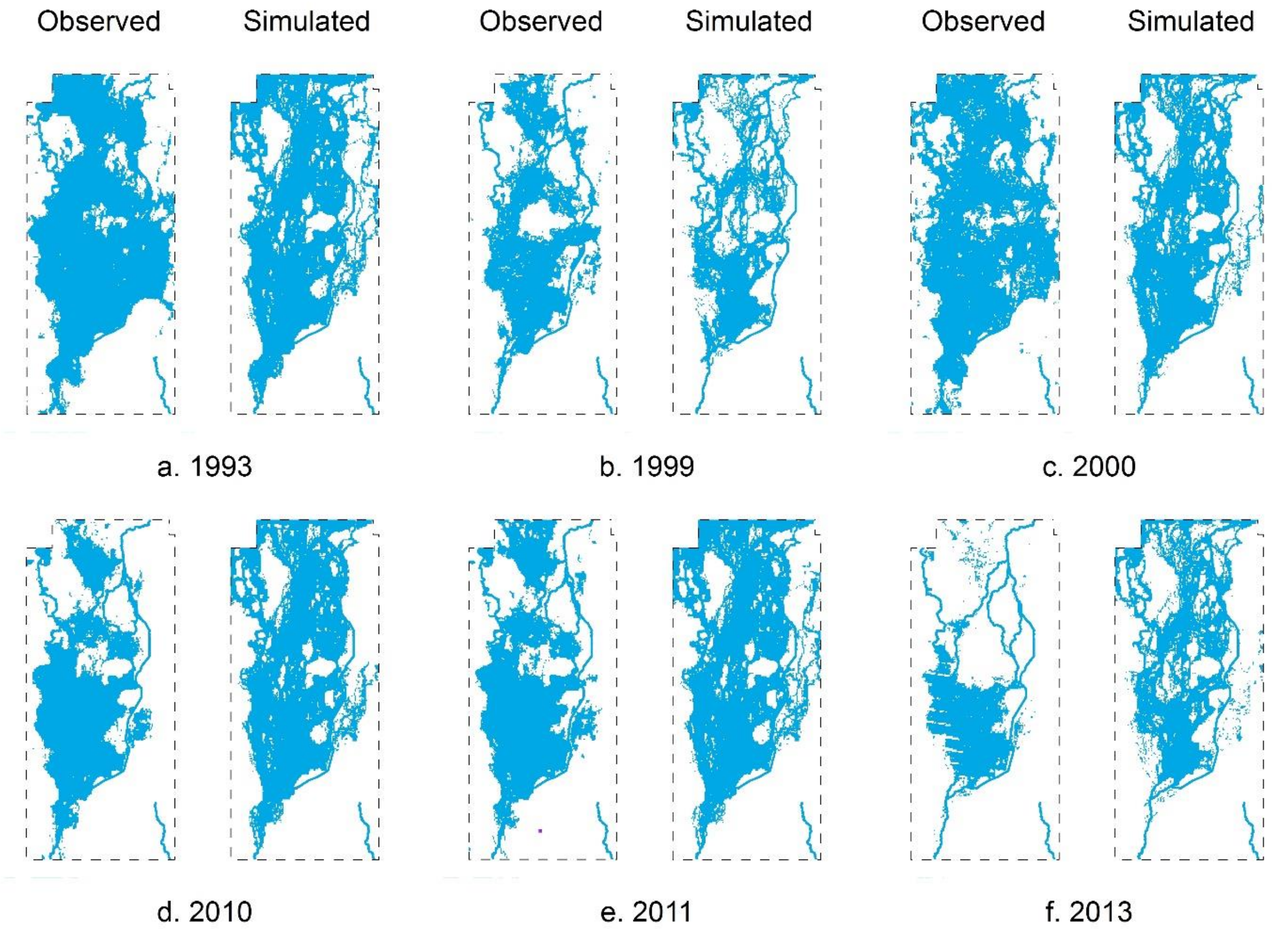

Fig. S3. Hydrodynamic model results – flood extent comparison. Comparison of observed and simulated inundation extent in the study site for six different events used for calibration and model testing. Performance indices presented in Table. S1.
Note: Adapted from Sandi et al. (2019).

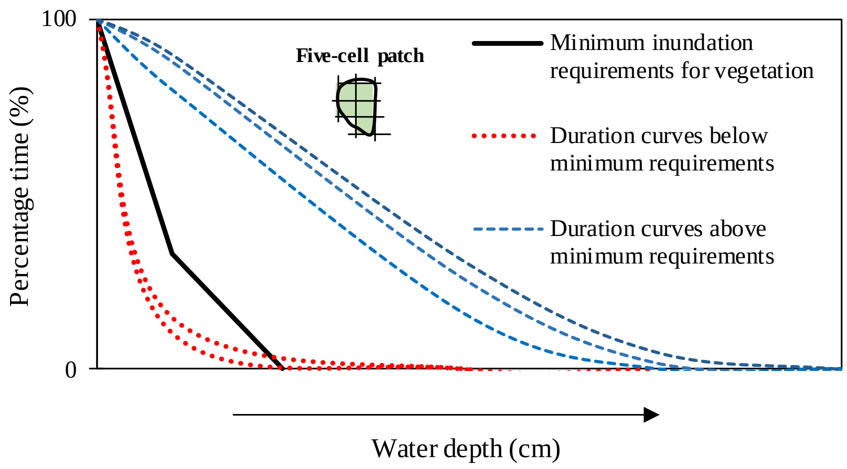

Fig. S4. Example of MII calculation for a five-cell patch. Each line in the figure represents the annual depth-duration curve for a given cell. Three out of five cells with annual depth-duration above the minimum threshold correspond to a MII of 60% in this patch.

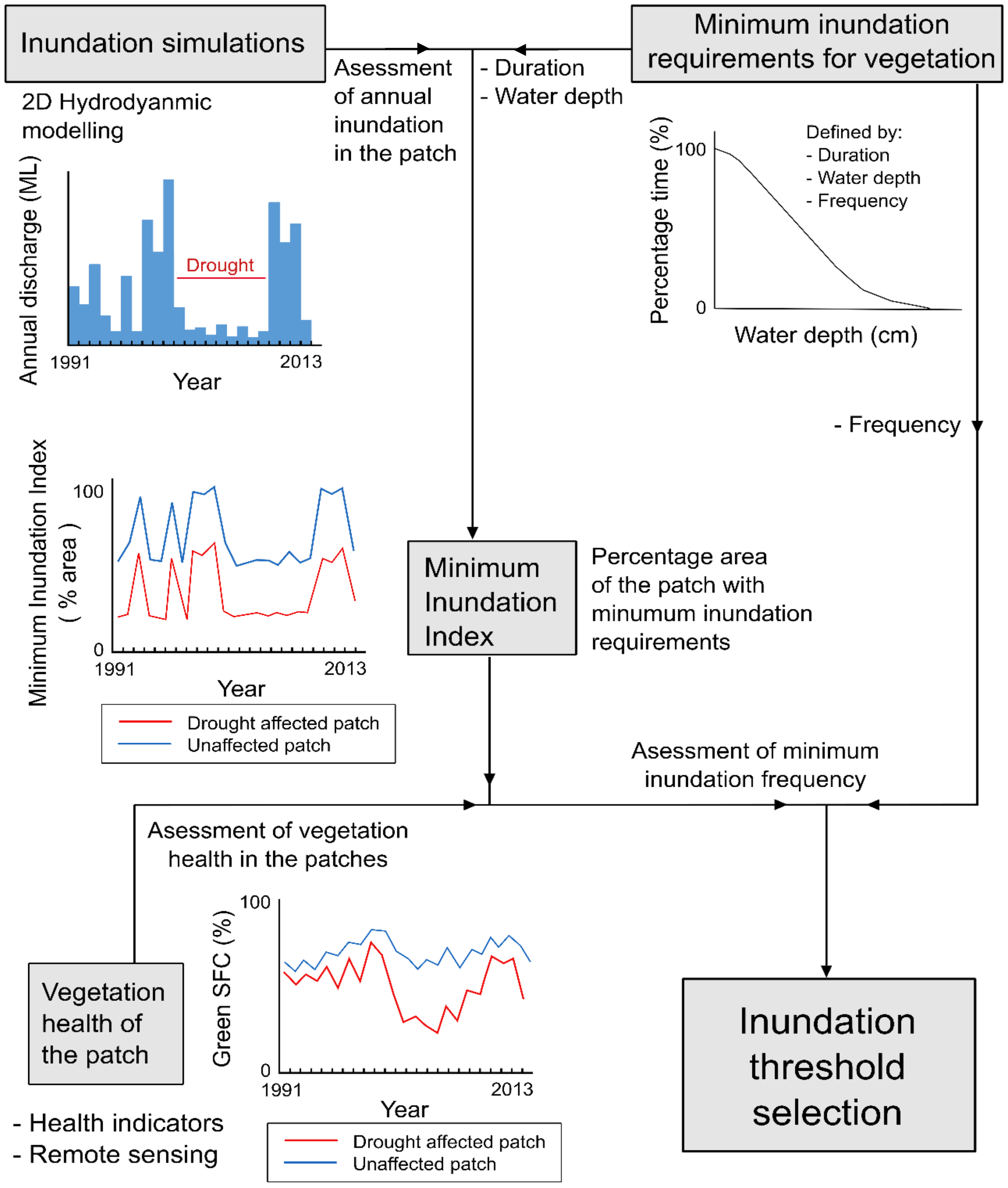

Fig. S5. Methodology scheme for obtaining MII thresholds for vegetation transition.
Note: Adapted from Sandi et al. (2019)

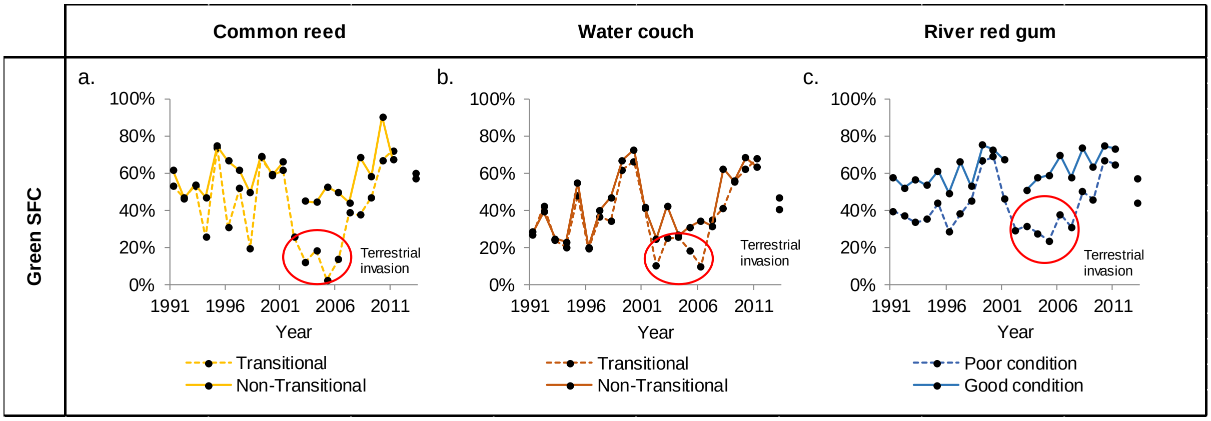

Fig. S6. Time series of green Seasonal Fractional Cover (SFC) for six selected patches. (**a**) Common Reed (**b**) Water Couch, and (**c**) River red gum patches show time of terrestrial invasion between 2003 and 2006, where green SFC reaches minimum values.

Note: Adapted from Sandi et al. (2019).

Table S1. Calibrated roughness values for each vegetation association in the study area

Land use and vegetation association	n<br>[$s \cdot m^{-1/3}$]
Channels	0.035 (clean) – 0.1 (vegetated)
Floodplain	
Cultivated	0.05
Terrestrial	0.036
Floodplain vegetation	0.053
River red gum association	0.07
Lignum	0.11
Water couch	0.075
Mixed marsh	0.057
Common reeds	0.11

Note: Table adapted from Sandi et al. (2019).

Table S2. Performance indices of the hydrodynamic model. Performance of the model for six different events obtained by comparing water depth recorded at station No.421151 and simulated inundation extent with remote sensing images. Performance indices include percent bias (PBIAS), Nash-Sutcliffe coefficient (NS) and the ratio of the root mean square error to the standard deviation of measured data (RSR).

Index	1993 event	1999 event	2000 event	2010 event	2011 event	2013 event
	N = 66<br>Model testing	N = 69<br>Calibration	N = 87<br>Model testing	N = 127<br>Calibration	N = 138<br>Calibration	N = 149<br>Model testing
**PBIAS***	10.0%	-0.3%	5.6%	5.6%	3.3%	-2.1%
**NS**	0.99	1.00	0.99	0.98	0.98	0.90
**RSR**	0.10	0.03	0.07	0.13	0.13	0.32
**Index**	**1993 event**	**1999 event**	**2000 event**	**2010 event**	**2011 event**	**2013 event**
**Overall Accuracy**	0.80	0.81	0.77	0.80	0.80	***
**Cohen's k**	0.60	0.59	0.54	0.59	0.59	

Note: *A negative PBIAS means overestimation and a positive PBIAS means underestimation. **N represents the number of days considered in each event. *** The 2013 inundation map was excluded due to low quality of the Landsat images. Tables adapted from Sandi et al. (2019).

Table S3. Inundation regime requirements for different plant associations

Plant Association	Duration (%)	Depth of water (m)	Frequency ($years^{-1}$)
Common reed	30% to 90%	0.02 to 0.5	0.33
Water couch	25% to 67%	0.02 to 0.6	0.33
River red gum	25% to 50%	0.02 to 0.6	0.14-0.2

Adapted from Sandi et al. (2019), based on Roberts and Marston (2011).

Table S4. Number of consecutive years of MII below threshold for River Red Gum.

River Red Gum condition	Number of consecutive years with MII below threshold from the last flood
Good*	1 or 2
Intermediate	3 to 6
Poor	7 or more

Note: *Good condition River Red Gum also requires at least two consecutive year with MII above threshold. Based on Roberts and Marston (2011).

Table S5. Range of MII thresholds tested during calibration of the vegetation model.

Vegetation	Required MII (% Area) for non-woody vegetation
Common Reed	10 – 65
Mixed Marsh/Water Couch	10 - 25
River Red Gum	10 - 60

Note: Sandi et al. (2019) reported the lower value of 10% for all vegetation associations. We have tested a larger range of thresholds for our simulations.

Table S6. List of GCMs from the CMIP5 downscaled for the climate change assessment by CSIRO and the Bureau of Meteorology.

CMIP5 Model ID	Institute	Country of Origin	Downscaling Method
ACCESS-1.0	CSIRO-BOM	Australia	Dynamic/Statistical
ACCESS-1.3	CSIRO-BOM	Australia	Statistical
BCC-CSM1-1-M	BCC, CMA	China	Statistical
BNU-ESM	BNU	China	Statistical
CanESM2	CCCMA	Canada	Statistical
CCSM4	NCAR	USA	Dynamic/Statistical
CMCC-CMS	CMCC	Italy	Statistical
CNRM-CM5	CNRM-CERFACS	France	Dynamic/Statistical
CSIRO-Mk3-6-0	CSIRO-QCCCE	Australia	Statistical
GFDL-CM3	NOAA, GFDL	USA	Dynamic
GFDL-ESM2G	NOAA, GFDL	USA	Statistical
GFDL-ESM2M	NOAA, GFDL	USA	Statistical
HadGEM2-CC	MOHC	UK	Statistical
IPSL-CM5A-LR	IPSL	France	Statistical
IPSL-CM5A-MR	IPSL	France	Statistical
IPSL-CM5B-LR	IPSL	France	Statistical
MIROC5	JAMSTEC	Japan	Statistical
MIROC-ESM	JAMSTEC	Japan	Statistical
MIROC-ESM-CHEM	JAMSTEC	Japan	Statistical
MPI-ESM-LR	MPI-N	Germany	Dynamic/Statistical
MPI-ESM-MR	MPI-N	Germany	Statistical
MRI-CGCM3	MRI	Japan	Statistical
NorESM1-M	NCC	Norway	Dynamic/Statistical

Note: Table based on data from CSIRO and BoM (2015).

References:

CSIRO & BOM 2015. Climate Change in Australia Projections for Australia's Natural Resource Management Regions: Technical Report. *In:* WHETTON, P., EKSTRÖM, M., GERBING, C., GROSE, M., BHEND, J., WEBB, L. & RISBEY, J. (eds.). Australia: CSIRO and Bureau of Meteorology.

ROBERTS, J. & MARSTON, F. 2011. *Water regime for wetland and floodplain plants: a source book for the Murray-Darling Basin,* Canberra, National Water Commission.

SANDI, S. G., SACO, P. M., SAINTILAN, N., WEN, L., RICCARDI, G., KUCZERA, G., WILLGOOSE, G. & RODRÍGUEZ, J. F. 2019. Detecting inundation thresholds for dryland wetland vulnerability. *Advances in Water Resources*, 128**,** 168-182.
